# System Justification Among the Disadvantaged: A Triadic Social Stratification Perspective

**DOI:** 10.3389/fpsyg.2020.00040

**Published:** 2020-01-31

**Authors:** Luca Caricati, Chuma K. Owuamalam

**Affiliations:** ^1^Department of Humanities, Social Sciences and Cultural Industries, University of Parma, Parma, Italy; ^2^Division of Organisational and Applied Psychology, University of Nottingham Malaysia Campus, Semenyih, Malaysia

**Keywords:** system justification theory, social identity model of system attitudes, triadic social stratification theory, system justification motivation, disadvantaged groups

The financial downturn in major economies of the world between 2007 and 2008 caused the bailout of several corporations and financial institutions that ostensibly served the economic interests of the wealthy 1% more than it did for the poorer 99%. Although there were pockets of resistance by the 99% (e.g., the occupy Wall Street movement), working- and middle-class people were surprisingly less supportive of economic redistributive policies and in favor of the prevailing economic order that squeezed the prospects of the less affluent more than it did the wealthy (Kuziemko et al., 2014; Jost, 2017; see also García-Sánchez et al., 2019). Elsewhere in the social psychological literature, research has documented a similar orientation amongst society's disadvantaged: the tendency to attribute more positive stereotypes/traits to privileged members of society, and often at the expense of their own group (the so-called “*outgroup favoritism effect*” Cichocka et al., 2015; Hoffarth and Jost, 2017; Samson, 2018).

Research that has tried to make sense of this paradoxical *system-justifying* outgroup favoritism has suggested that such an effect may be more pronounced when status differences between the disadvantaged and the advantaged are seen as legitimately achieved, and when the system is perceived to be inescapable and durable/stable (Friesen et al., 2019). Indeed, the outgroup favoritism phenomenon is described as “system-justifying” because such tendencies have the potential to entrench social inequality, especially when these attitudes are held by people who are disadvantaged in the prevailing order. This evidence of system-justifying attitudes among disadvantaged appears puzzling because these are people who incur several psychological costs (such as reduced collective self-esteem and entitlement, and increased psychological maladjustment see Major, 1994; Jetten et al., 2017) by virtue of their poorer outcomes within existing societal arrangements. That is, one would have expected (e.g., from a rational choice perspective, Coleman, 1990) for the less privileged in society to be more supportive of systems and policies than serve their interest, rather than those that ostensibly strip them away (Riker and Ordeshook, 1968; Feddersen, 2004).

Different theoretical formulations have largely focused on *when* the puzzling occurrence of system-justifying attitudes is most likely (Friesen et al., 2019), especially amongst society's disadvantaged (Jost, 2017, 2019). However, the unfolding debate around the phenomenon now centers on *why* the disadvantaged would hold such attitudes in the first place. In this opinion paper, we consider the dominant perspective put forward by the system justification theory (Jost and Banaji, 1994), and then contrast its explanation with alternative propositions, including the newer triadic social stratificationtheory (Caricati, 2018).

## Explaining the System Justification Effect via the System Justification Theory

The system justification theory (SJT; Jost and Banaji, 1994) recognizes—as do other perspectives like social identity theory (Tajfel and Turner, 1979)—that people are motivated to support their self (ego) and group interests. However, SJT goes further to propose the existence of an autonomous motivation that supports the existing social arrangement, called the *system justification motivation*. According to SJT, people are driven by a conscious or unconscious system-oriented need “to defend, bolster, and justify existing social, economic, and political institutions and arrangements” (Jost and Kay, 2010, p. 1,148) and this represents a further type of human motivation because it functions to support the status quo alone (Jost and Banaji, 1994, p. 10). According to the original formulation of SJT (Jost and Banaji, 1994) and its subsequent refinements (e.g., Jost et al., 2004), this system-oriented motivation is ostensibly rooted in epistemic needs (e.g., to avoid uncertainty), existential needs (e.g., to reduce distress and threat), and relational needs (e.g., to embrace shared realities; Jost et al., 2008), which manifests most strongly when people's yearnings for predictability and/or certainty within a system that they depend on, is strong (Jost, 2017). Given that the stability and predictability of existing systems guarantees the benefits (or interests) of the privileged, it is cognitively straightforward for society's advantaged to support societal systems that ensures their privileged position. However, supporting unequal societal systems may not be as straightforward for society's disadvantaged (i.e., the 99%) as it might be for their advantaged counterparts. According to SJT, this is because, for the disadvantaged, satisfying their inner yearning for predictability (and control) via support for existing arrangements may come at the expense of relinquishing their struggle for equity/equality (i.e., group interests), and these competing demands are likely to cause cognitive dissonance (Festinger, 1957)—a psychological dilemma that people are often motivated to eliminate/avoid.

Hence, SJT contends that acquiescing to the status-quo may be a much easier strategy for the disadvantaged to resolve their cognitive dilemma, than to adopt the potentially uphill task of changing (legitimate and stable) realities that people have become accustomed to Jost et al. (2012). According to SJT, this scenario creates the potential for the disadvantaged to be even more likely than their privileged counterparts to justify disadvantageous realities because, such rationalization can help to soothe the pain associated with their discomforting internal struggle (Jost and Hunyday, 2002; Osborne and Sibley, 2013; c.f. Owuamalam et al., 2017). In short, according to SJT, the disadvantaged support societal systems/tradition because a system justification motive that operates in the opposite direction to people's interests causes them to do so.

## How Strong Is the Evidential Basis for SJT's Dissonance-Inspired Explanation for the System Justification Effect?

Consistent with SJT, pockets of nationally representative cross-sectional surveys (e.g., Jost et al., 2003; Henry and Saul, 2006; Sengupta et al., 2015), and experimental studies (e.g., van der Toorn et al., 2015) have shown that the disadvantaged may support societal systems more strongly than their privileged counterparts do, especially when they are dependent on such systems. However, an even greater number of similar nationally representative surveys (Caricati and Lorenzi-Cioldi, 2012; Brandt, 2013; Caricati, 2017; Vargas-Salfate et al., 2018; see Yang et al., 2019 for a review) have reported unsupportive evidence for the dissonance-inspired version of the system justification thesis, showing that system justification increases as social advantage increases. The unsurportive evidence for SJT's dissonance-inspired explanation is not limited to cross-sectional studies. Experimental studies also report contradictory evidence (e.g., Trump and White, 2018; Owuamalam and Spears, 2020), even when a sense of poverty (vs. affluence) is experimentally induced: people tend to show a greater inclination toward challenging unequal systems by, for example, a fair allocation of rewards to the relevant parties (Bratanova et al., 2016). Other indirect evidence corroborate the foregoing trends, showing that the disadvantage (e.g., African Americans) are more likely to endorse the conspiratorial belief that the system is rigged against African Americans (Crocker et al., 1999), when a standard reading of SJT would suggest otherwise.

## Criticisms and Other Explanations for the System Justification Effect

In the face of the foregoing empirical discrepancies (see also Li et al., 2020), Owuamalam et al. (2018, 2019a,b) have queried the necessity of SJT's system motive explanation and proposed instead that the system justification effect can be more parsimoniously explained with the traditional interest-based perspectives via their social identity model of system attitudes (SIMSA). Rooted in the social identity tradition, SIMSA assumes that the system justification effect can be driven by the need for accuracy and a positive social identity, and advances three explanations in these regards. The first explanation is that, when positions within an existing order are legitimate and stable, system-justifying attitudes can occur amongst the disadvantaged because accuracy motives constraint their ability to objectively contest the superiority of a clearly superior outgroup competitor. The second explanation is that, when the system is unstable in the long run, system justifying attitudes can represent an expression of hope that the system will one day provide the opportunity for the upward advancement of one's disadvantaged ingroup. The third explanation is that, when an inclusive social identity is salient, system justification effect can result from ingroup bias at this superordinate level of self-categorization, such that system support is nothing more than an expression of common-ingroup favoritism.

Although SIMSA as a theoretical framework for understanding the system justification effect is in its nascent stages, available evidence corroborates some of its key assumptions. For example, some studies have shown a positive correlation between system justification and hope for both the future advancement of the ingroup (Owuamalam et al., 2016; Sollami and Caricati, 2018; see also Vasilopoulos and Brouard, 2019) and individual mobility (Li et al., 2019). Others have shown that members of a religious minority group who emphasized their inclusive (common-ingroup) identity (e.g., their nation) reported stronger system-justifying attitudes (Jaśko and Kossowska, 2013). In short, consistent with SIMSA's explanations, there is evidence that the system justification effect might be the disadvantaged's attempt to defend, protect and bolster their social identity.

## The Triadic Social Stratification Explanation for the System Justification Effect

The triadic social stratification theory (TSST; Caricati, 2018) agrees with SIMSA in proposing that the system justification effect can be rooted in social identity needs. However, unlike SIMSA (or SJT for that matter), TSST focuses on processes of intergroup comparison that can help to explain the system justification effect amongst disadvantaged groups within a triadic (even multiple) hierarchical system. The key assumption here is that, in several social hierarchies, groups are neither inherently high in status (e.g., the 1%) or low in status (e.g., the 99%), and that disadvantage (vs. advantage) often depend on the existence of one or more status outgroups to which one's group compares on some material, psychological or social outcome. Because people are motivated to achieve a positive social identity, there is often the tendency to engage in intergroup comparisons that maximize people's chances of achieving this goal. Members of intermediately positioned disadvantaged groups might compare their outcomes to those who are worse-off than they are (i.e., downward comparison) rather than better-off than they are (i.e., upward comparison), and this type of contrast can enable a sense of positive identity (and satisfaction) needed to accept the way things are (Dunham et al., 2014).

But, intermediately placed groups are still lower in status to group(s) that are higher-up in the social stratification, and it is possible that both downward (favorable) and upward (unfavorable) comparisons may be simultaneously active sometimes (e.g., Caricati, 2012), and how system justification is navigated under such circumstance becomes important. Of course the system justification effect is unlikely to emerge when upward (unfavorable) comparison trumps downward (favorable) comparison, and this provision helps to explain a range of radical and non-radical demonstrations of discontent that are seen amongst the disadvantaged (Wright, 2009; Teixeira et al., 2019). Our point, however, is that so long as downward (favorable) comparisons overwhelm the potential for unfavorable comparisons, system justification should be a likely outcome amongst the disadvantaged. In short, the flexibility in the choice of intergroup comparison amongst intermediately placed disadvantaged groups, can provide the incentive for supporting the status quo because, at some level, the existing reality isn't as bad for them as it is for other groups that are lower down the “food chain” (Becker, 2012). That is, if disadvantaged groups can achieve a positive identity via downward comparison(s), they may be motivated to support a system in order to protect the interests that are already satisfied by an arrangement that affords them more opportunities than others. Supportive evidence for this argument comes from Caricati and Sollami (2018), showing that nurses were more likely to justify the hierarchically sorted healthcare professional system when they could compare their outcomes to those of their lower status counterparts (i.e., healthcare assistants) relative to when this favorable downward comparison was not possible.

## Comparisons Across Time

**Figure 1:**
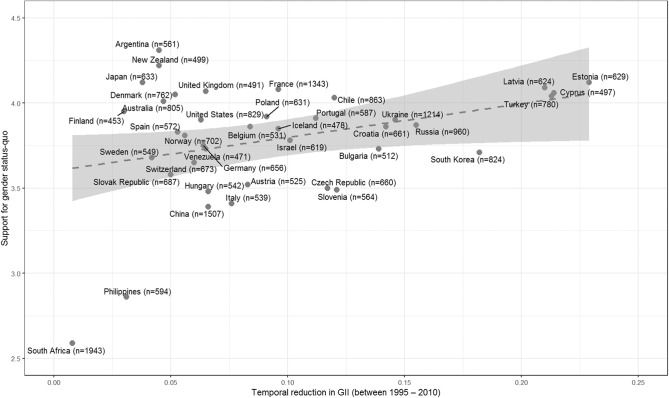
Degree of reduction in gender inequality between 1995 and 2010 predicts tacit support for the gender status-quo in 2009 amongst 27,970 women in 39 nations, *r* = 0.33 (*N* = 39, *p* = 0.04) (ISSP Research Group, 2017) (source: ISSP Research Group, 2017). Gender inequality index (GII; United Nations Development Programme, 2019) measures the inequality in achievement between women and men in three dimensions: reproductive health, empowerment and the labor market. Support for gender status-quo was measured with the item “Getting ahead: How important is being born a man or a woman?” (1 = *essential*, 5 =*Not important at all);* because accepting that gender does not matter in getting ahead represents satisfaction with the gender status-quo. This item is also conceptually similar to other items on the gender system justification measure [e.g., “everyone (male or female) has a fair shot and wealth and happiness”—Jost and Kay, 2005]. Gender is conceived here, not as a binary category, but as a multi- layered social stratification that includes men, women, and then transgendered people.

The foregoing comparison-based explanation relates to a single time point (i.e., the justification of an *existing* social arrangement). It is also possible to conceive of situations in which comparisons can be made across different time points, such as when people compare their present with their past (e.g., Zagefka and Brown, 2005; Guimond and de la Sablonnière, 2015), their future (Owuamalam et al., 2018) or their temporal intergroup outcomes (de la Sablonnière et al., 2009; Bougie et al., 2011). TSST assumes that as long as these temporal comparisons are favorable (in the present or future), system justification should be a likely outcome amongst members of intermediately placed disadvantaged groups because, they are distinctly enabled by their uniquely malleable position to exploit fluctuations within the system. That is, intermediately placed disadvantaged groups might believe that the existing system is fair (and justified) because it has permitted an improvement to their group's position relative to its situation in the past, or because it will permit further improvements to their outcomes in the future (akin to Owuamalam et al., 2018 hope for future ingroup status explanation). Although evidence for this latter proposition is absent in the published literature, other publicly archived data from the International Social Survey Program (ISSP) provide an initial confirmation of these assumptions. As Figure 1 indicates, women in countries where the gender inequality index (GII) has reduced considerably in 2010 from what it was in the past (down to 1995), tend to be more supportive of the gender status-quo—dismissing the notion that gender is a relevant factor for upward social mobility.

## Concluding Remarks

To be clear, we are neither proposing a general theory of intergroup relations, nor is the goal here to explain all instances of system justification amongst the disadvantaged. Rather, our aim was to use insights from the TSST to offer a new identity-based explanation for the system justification effect among society's disadvantaged. Indeed, the dominant explanation for the system justification effect has been the assumption of a system motive that runs counter to self/group interests. However, both proponents and opponents of this “special system motive” explanation do not neatly account for the effect of intergroup comparisons on system justification. We close this gap by proposing that instances of system justification among the disadvantaged can also be traced back to the favorable comparisons that are possible when disadvantaged groups occupy an intermediate position within a multiple stratified status system. Furthermore, the current analysis extends these insights to temporal comparisons, and suggests that system justification is likely to manifest amongst intermediately placed disadvantaged groups when these (temporal) contrasts are favorable.

Finally, it is tempting to argue, based on SJT, that intergroup comparisons may be part-and-parcel of the dissonance process that causes system-justifying tendencies amongst the disadvantaged because, it potentially involves the *suppression* of an upward comparison that ordinarily enables group-based motives, while at the same time *permitting* a downward comparison that should allow the system motive to thrive. The problem with this argumentation, however, is that it becomes difficult to separate the effects that are tied to the system motive from an interest-based explanation because, in this situation, intermediately positioned disadvantaged group members may be supporting the status quo because they are at least better-off than others. Research is needed to unpack these complexities.

## Author Contributions

All authors listed have made a substantial, direct and intellectual contribution to the work, and approved it for publication.

### Conflict of Interest

The authors declare that the research was conducted in the absence of any commercial or financial relationships that could be construed as a potential conflict of interest.
